# (2-{[2-Carboxyl­ato-1-(4-chloro­phen­yl)eth­yl]imino­meth­yl}phenolato-κ^3^ *O*,*N*,*O*′)(1*H*-imidazole-κ*N* ^3^)copper(II) monohydrate

**DOI:** 10.1107/S1600536810014765

**Published:** 2010-04-28

**Authors:** Wen-Jun Zhou, Yin-Zhi Jiang, Yang Zou

**Affiliations:** a Chemistry Department, Zhejiang Sci-Tech University, Hangzhou 310018, People’s Republic of China

## Abstract

The Cu^II^ atom of the title complex, [Cu(C_16_H_12_ClNO_3_)(C_3_H_4_N_2_)]·H_2_O, has a distorted square-planar coordination geometry formed by a tridentate Schiff base dianion and an imidazole ligand. The imidazole is nearly coplanar with the coordination plane, the dihedral angle between the planes being 3.73 (12)°. In the Schiff base ligand, the two benzene rings are oriented at a dihedral angle of 75.87 (12)°. O—H⋯O and N—H⋯O hydrogen bonding is present in the crystal structure. One H atom of the uncoordinated water mol­ecule is disordered equally over two sites.

## Related literature

Transition metal complexes of salicylaldehyde-peptides and salicylaldehyde-amino­acid Schiff bases are non-enzymatic models for pyridoxal amino acid systems, which are of importance as key inter­mediates in many metabolic reactions of amino acid catalyses by enzymes, see: Bkouche-Waksman *et al.* (1988 ▶); Wetmore *et al.* (2001 ▶); Zabinski & Toney (2001 ▶). For the preparation, structural characterization, appropriate spectroscopy and magnetic studies of Schiff-base complexes derived from salicylaldehyde and amino acids, see: Ganguly *et al.* (2008 ▶) and references cited therein. For Schiff bases derived from *β*-amino acids, see: Vančo *et al.* (2008 ▶).
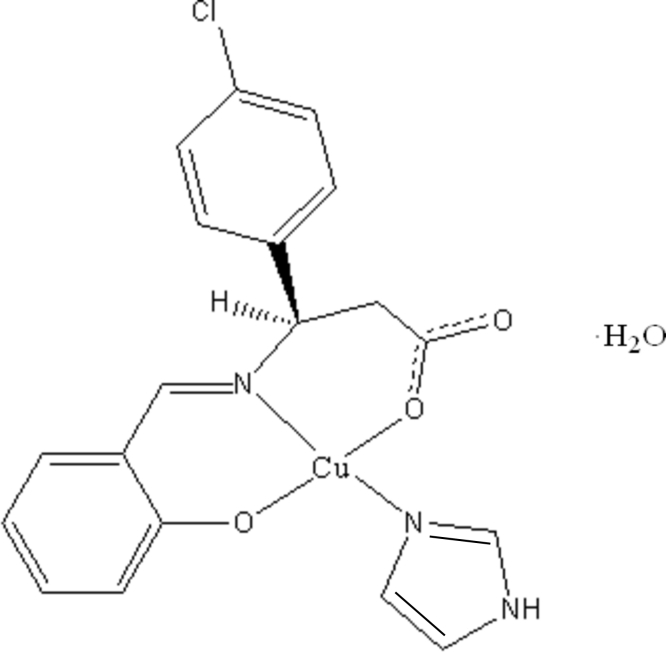


## Experimental

### 

#### Crystal data

[Cu(C_16_H_12_ClNO_3_)(C_3_H_4_N_2_)]·H_2_O
*M* *_r_* = 451.35
Monoclinic, 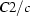

*a* = 23.884 (1) Å
*b* = 4.944 (1) Å
*c* = 32.008 (1) Å
β = 96.88 (1)°
*V* = 3752.4 (8) Å^3^
*Z* = 8
Mo *K*α radiation
μ = 1.34 mm^−1^
*T* = 296 K
0.20 × 0.20 × 0.15 mm

#### Data collection

Bruker SMART CCD diffractometer
Absorption correction: multi-scan (*SADABS*; Bruker, 2003 ▶) *T*_min_ = 0.776, *T*_max_ = 0.825
18085 measured reflections
4310 independent reflections
3298 reflections with *I* > 2σ(*I*)
*R*_int_ = 0.035

#### Refinement

*R*[*F* ^2^ > 2σ(*F* ^2^)] = 0.035
*wR*(*F* ^2^) = 0.087
*S* = 1.04
4310 reflections
253 parameters
H-atom parameters constrained
Δρ_max_ = 0.33 e Å^−3^
Δρ_min_ = −0.30 e Å^−3^

### 

Data collection: *SMART* (Bruker, 2003 ▶); cell refinement: *SAINT* (Bruker, 2003 ▶); data reduction: *SAINT*; program(s) used to solve structure: *SHELXTL* (Sheldrick, 2008 ▶); program(s) used to refine structure: *SHELXTL*; molecular graphics: *SHELXTL*; software used to prepare material for publication: *SHELXTL*.

## Supplementary Material

Crystal structure: contains datablocks I, global. DOI: 10.1107/S1600536810014765/xu2751sup1.cif

Structure factors: contains datablocks I. DOI: 10.1107/S1600536810014765/xu2751Isup2.hkl

Additional supplementary materials:  crystallographic information; 3D view; checkCIF report

## Figures and Tables

**Table 1:** Selected bond lengths (Å)

Cu1—O1	1.8894 (16)
Cu1—O3	1.9494 (16)
Cu1—N1	1.9582 (18)
Cu1—N2	1.9789 (18)

**Table 2:** Hydrogen-bond geometry (Å, °)

*D*—H⋯*A*	*D*—H	H⋯*A*	*D*⋯*A*	*D*—H⋯*A*
O1*W*—H1*W*⋯O2^i^	0.87	1.98	2.799 (3)	156
O1*W*—H2*W*1⋯O1*W*^ii^	0.82	2.01	2.826 (4)	172
O1*W*—H2*W*2⋯O1*W*^iii^	0.83	2.02	2.822 (5)	163
N3—H3*A*⋯O2^iv^	0.86	1.90	2.758 (3)	172

Symmetry codes: (i) 
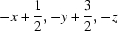
; (ii) 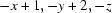; (iii) 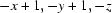; (iv) 
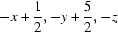
.

**Fig. 1.**
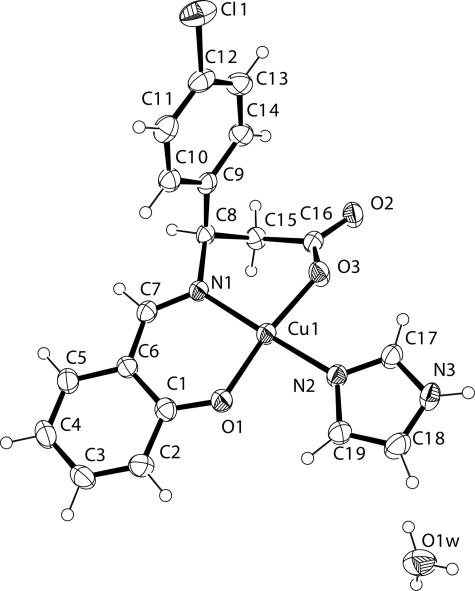
The structure of the title complex with atom numbering scheme; thermal ellipsoids are drawn at 40% probability level.

**Fig. 2.**
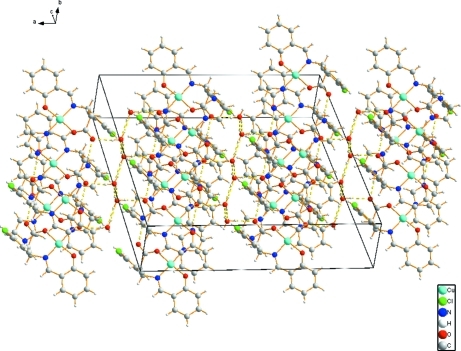
Schematic representation of the hydrogen-bonded (dashed lines).

## supplementary crystallographic information

### Comment

Transition metal complexes of salicylaldehyde-peptides and salicylaldehyde-amino acid Schiff base are non-enzymatic models for pyridoxal-amino acid systems, which are of considerable importance as key intermediates in many metabolic reactions of amino acids catalyzed by enzymes (Zabinski *et al.*, 2001; Wetmore *et al.*, 2001; Bkouche-Waksman *et al.*,1988). Considerable effort has been devoted to the preparation, structural characterization, appropriate spectroscopy and magnetic studies of Schiff-base complexes derived from salicylaldehyde and amino acids and reduced salicylidene amino acid (Ganguly *et al.*, 2008), but little attention has been given to Schiff base derived from *β*-amino acid (Vančo *et al.*, 2008). Herein, we report the structure study of [Cu(L)(C_3_H_4_N_2_)]^. ^H_2_O (H_2_L= Schiff bases derived from glycylglycine and salicylaldehyde, C_16_H_14_NO_3_Cl).

The complex crystallizes in the monoclinic space group C2/c. The title molecule,is characterized by a square-planar Cu^II^ coordination with the deprotonated tridentate Schiff base dianion and one imidazole molecule in the basal plane (Fig. 1). The Cu1—N1 bond distance is 1.958 Å. The two Cu—O bonds are 1.889(Cu1—O1) and 1.950 Å (Cu1—O3). The fourth position occupied by one N atom from the imidazole ligand, with bond length of 1.980 Å (Cu1—N2). The phenyl ring [C1—C6] and the ring of C1, C6, C7, N1, O1, Cu1 chelate ring are almost coplanar with a small dihedral angle of 1.8^o^. Hydrogen bond between the coordinated imidazole molecule and the carboxyl oxygen atom of an adjacent, symmetry related CuL unit leads to the formation of a [CuL(C_3_H_4_N_2_)]_2_ dimer. Hydrogen bond between water molecule and CuL unit further link the dimers into two-dimension layers (Fig. 2).

### Experimental

The Schiff base was prepared through the condensation of 3-amino-3-(4-chlorophenyl) propionic acid and salicylaldehyde. 3-Amino-3-(4-chlorophenyl) propionic acid (10 mmol) was dissolved and refluxed in absolute methanol (40 ml) containing LiOH^.^H_2_O (10 mmol). After cooled to room temperature, a solution of salicylaldehyde (10 mmol) in absolute methanol was added slowly with stirring over 10 min. Then Cu(NO_3_)_2_ (10 mmol) was added to the HLLi solution and the resulting solution was adjusted to the pH = 9-11 by 1.0 mol/L NaOH solution. After stirring at room temperature for 30 min, imidazole (10 mmol) was added to the solution with stirring. The resulting clear solution was then filtered. The filtrate was allowed to evaporate slowly at room temperature. After several days dark green crystals suitable for X-ray diffraction were obtained.

### Refinement

One H atom of the lattice water molecule is equally disordered over two sites. The water H atoms were placed in chemical sensitive positions and refined with distance restraint of O—H = 0.85 Å and U_iso_(H) = 1.2U_eq_(O). Other H atoms were positioned geometrically and constrained as riding atoms with C—H = 0.93–0.98 Å and N—H = 0.86 Å, U_iso_(H) = 1.2U_eq_(C,N).

### Crystal data

**Table:**

[Cu(C_16_H_12_ClNO_3_)(C_3_H_4_N_2_)]·H_2_O	*F*(000) = 1848
*M**_r_* = 451.35	*D*_x_ = 1.598 Mg m^−^^3^
Monoclinic, *C*2/*c*	Mo *K*α radiation, λ = 0.71073 Å
Hall symbol: -C 2yc	Cell parameters from 3298 reflections
*a* = 23.884 (1) Å	θ = 1.7–27.5°
*b* = 4.944 (1) Å	µ = 1.34 mm^−^^1^
*c* = 32.008 (1) Å	*T* = 296 K
β = 96.88 (1)°	Block, dark green
*V* = 3752.4 (8) Å^3^	0.20 × 0.20 × 0.15 mm
*Z* = 8	

### Data collection

**Table:**

Bruker SMART CCD diffractometer	4310 independent reflections
Radiation source: fine-focus sealed tube	3298 reflections with *I* > 2σ(*I*)
graphite	*R*_int_ = 0.035
φ and ω scans	θ_max_ = 27.5°, θ_min_ = 1.7°
Absorption correction: multi-scan (*SADABS*; Bruker, 2003)	*h* = −30→30
*T*_min_ = 0.776, *T*_max_ = 0.825	*k* = −6→6
18085 measured reflections	*l* = −41→41

### Refinement

**Table:**

Refinement on *F*^2^	Primary atom site location: structure-invariant direct methods
Least-squares matrix: full	Secondary atom site location: difference Fourier map
*R*[*F*^2^ > 2σ(*F*^2^)] = 0.035	Hydrogen site location: inferred from neighbouring sites
*wR*(*F*^2^) = 0.087	H-atom parameters constrained
*S* = 1.04	*w* = 1/[σ^2^(*F*_o_^2^) + (0.0381*P*)^2^ + 2.3614*P*] where *P* = (*F*_o_^2^ + 2*F*_c_^2^)/3
4310 reflections	(Δ/σ)_max_ < 0.001
253 parameters	Δρ_max_ = 0.33 e Å^−^^3^
0 restraints	Δρ_min_ = −0.29 e Å^−^^3^

### Special details

**Table:**

Geometry. All esds (except the esd in the dihedral angle between two l.s. planes) are estimated using the full covariance matrix. The cell esds are taken into account individually in the estimation of esds in distances, angles and torsion angles; correlations between esds in cell parameters are only used when they are defined by crystal symmetry. An approximate (isotropic) treatment of cell esds is used for estimating esds involving l.s. planes.
Refinement. Refinement of F^2^ against ALL reflections. The weighted R-factor wR and goodness of fit S are based on F^2^, conventional R-factors R are based on F, with F set to zero for negative F^2^. The threshold expression of F^2^ > 2sigma(F^2^) is used only for calculating R-factors(gt) etc. and is not relevant to the choice of reflections for refinement. R-factors based on F^2^ are statistically about twice as large as those based on F, and R- factors based on ALL data will be even larger.

### Fractional atomic coordinates and isotropic or equivalent isotropic displacement parameters (Å^2^)

**Table:**

	*x*	*y*	*z*	*U*_iso_*/*U*_eq_	Occ. (<1)
Cu1	0.257392 (11)	0.70003 (6)	0.103939 (8)	0.03102 (10)	
Cl1	−0.00394 (3)	1.30201 (17)	0.18252 (3)	0.0645 (2)	
N1	0.20142 (7)	0.4707 (4)	0.12667 (6)	0.0301 (4)	
N2	0.31243 (7)	0.9444 (4)	0.08174 (6)	0.0332 (4)	
N3	0.34883 (9)	1.2624 (4)	0.04560 (7)	0.0414 (5)	
H3A	0.3513	1.3915	0.0279	0.050*	
O1	0.31821 (6)	0.5542 (4)	0.14014 (5)	0.0406 (4)	
O2	0.13208 (7)	0.8259 (4)	0.00906 (5)	0.0425 (4)	
O3	0.20152 (7)	0.8490 (4)	0.06065 (5)	0.0436 (4)	
C1	0.31811 (10)	0.3666 (5)	0.16842 (7)	0.0351 (5)	
C2	0.36998 (10)	0.2833 (6)	0.19110 (8)	0.0439 (6)	
H2	0.4033	0.3671	0.1858	0.053*	
C3	0.37183 (11)	0.0827 (6)	0.22041 (7)	0.0447 (6)	
H3	0.4065	0.0316	0.2346	0.054*	
C4	0.32307 (11)	−0.0477 (6)	0.22958 (8)	0.0462 (7)	
H4	0.3250	−0.1838	0.2498	0.055*	
C5	0.27261 (10)	0.0272 (5)	0.20854 (7)	0.0409 (6)	
H5	0.2400	−0.0594	0.2146	0.049*	
C6	0.26851 (10)	0.2331 (5)	0.17774 (7)	0.0329 (5)	
C7	0.21439 (10)	0.2914 (5)	0.15579 (7)	0.0335 (5)	
H7	0.1846	0.1879	0.1633	0.040*	
C8	0.14124 (9)	0.4717 (5)	0.10794 (7)	0.0308 (5)	
H8	0.1251	0.2993	0.1157	0.037*	
C9	0.10709 (9)	0.6933 (5)	0.12577 (7)	0.0313 (5)	
C10	0.11509 (10)	0.7554 (5)	0.16848 (8)	0.0383 (6)	
H10	0.1440	0.6701	0.1857	0.046*	
C11	0.08136 (10)	0.9405 (6)	0.18610 (8)	0.0433 (6)	
H11	0.0875	0.9788	0.2147	0.052*	
C12	0.03856 (10)	1.0673 (5)	0.16069 (8)	0.0426 (6)	
C13	0.02920 (10)	1.0118 (5)	0.11820 (8)	0.0431 (6)	
H13	0.0003	1.0987	0.1013	0.052*	
C14	0.06307 (9)	0.8260 (5)	0.10105 (8)	0.0387 (6)	
H14	0.0565	0.7881	0.0724	0.046*	
C15	0.13857 (9)	0.4720 (5)	0.05973 (7)	0.0331 (5)	
H15A	0.1617	0.3250	0.0513	0.040*	
H15B	0.1000	0.4373	0.0477	0.040*	
C16	0.15803 (9)	0.7320 (5)	0.04156 (7)	0.0325 (5)	
C17	0.30207 (10)	1.1329 (5)	0.05273 (7)	0.0385 (6)	
H17	0.2664	1.1707	0.0389	0.046*	
C18	0.39202 (11)	1.1523 (6)	0.07154 (8)	0.0487 (7)	
H18	0.4298	1.2016	0.0735	0.058*	
C19	0.36968 (9)	0.9583 (6)	0.09385 (8)	0.0408 (6)	
H19	0.3897	0.8504	0.1142	0.049*	
O1W	0.48323 (8)	0.7498 (5)	0.01584 (7)	0.0703 (6)	
H1W	0.4469	0.7337	0.0159	0.084*	
H2W1	0.4959	0.8883	0.0066	0.084*	0.50
H2W2	0.4987	0.6209	0.0050	0.084*	0.50

### Atomic displacement parameters (Å^2^)

**Table:**

	*U*^11^	*U*^22^	*U*^33^	*U*^12^	*U*^13^	*U*^23^
Cu1	0.02878 (15)	0.03081 (17)	0.03347 (16)	−0.00405 (12)	0.00374 (11)	0.00462 (13)
Cl1	0.0498 (4)	0.0640 (5)	0.0836 (6)	0.0023 (4)	0.0240 (4)	−0.0184 (4)
N1	0.0281 (9)	0.0298 (11)	0.0323 (10)	−0.0028 (8)	0.0034 (8)	0.0038 (9)
N2	0.0315 (10)	0.0363 (12)	0.0314 (10)	−0.0053 (9)	0.0027 (8)	0.0040 (9)
N3	0.0459 (12)	0.0394 (13)	0.0399 (11)	−0.0119 (10)	0.0094 (9)	0.0071 (10)
O1	0.0320 (8)	0.0453 (11)	0.0439 (10)	−0.0041 (8)	0.0017 (7)	0.0127 (9)
O2	0.0417 (9)	0.0455 (11)	0.0387 (9)	−0.0073 (8)	−0.0022 (8)	0.0123 (8)
O3	0.0362 (9)	0.0392 (11)	0.0525 (10)	−0.0121 (8)	−0.0064 (8)	0.0160 (9)
C1	0.0399 (13)	0.0362 (14)	0.0290 (12)	0.0020 (11)	0.0040 (10)	−0.0005 (11)
C2	0.0349 (13)	0.0546 (18)	0.0418 (14)	0.0031 (12)	0.0025 (11)	0.0028 (13)
C3	0.0447 (14)	0.0524 (17)	0.0354 (13)	0.0139 (13)	−0.0020 (11)	0.0034 (13)
C4	0.0584 (16)	0.0458 (17)	0.0340 (13)	0.0074 (14)	0.0034 (12)	0.0102 (12)
C5	0.0450 (14)	0.0396 (15)	0.0381 (13)	−0.0007 (12)	0.0050 (11)	0.0069 (12)
C6	0.0354 (12)	0.0322 (13)	0.0310 (12)	0.0030 (10)	0.0044 (9)	0.0014 (10)
C7	0.0341 (12)	0.0310 (13)	0.0364 (12)	−0.0047 (11)	0.0081 (10)	0.0022 (11)
C8	0.0276 (11)	0.0297 (12)	0.0349 (12)	−0.0080 (10)	0.0026 (9)	0.0058 (10)
C9	0.0271 (11)	0.0332 (13)	0.0340 (12)	−0.0093 (10)	0.0043 (9)	0.0028 (11)
C10	0.0343 (12)	0.0436 (16)	0.0366 (13)	−0.0048 (11)	0.0023 (10)	0.0048 (11)
C11	0.0448 (14)	0.0499 (17)	0.0359 (13)	−0.0082 (13)	0.0076 (11)	−0.0029 (13)
C12	0.0344 (12)	0.0404 (15)	0.0552 (16)	−0.0069 (12)	0.0152 (11)	−0.0048 (13)
C13	0.0293 (12)	0.0477 (16)	0.0508 (15)	−0.0029 (12)	−0.0009 (11)	0.0003 (13)
C14	0.0329 (12)	0.0457 (16)	0.0367 (13)	−0.0029 (12)	0.0003 (10)	−0.0016 (12)
C15	0.0348 (12)	0.0282 (13)	0.0355 (12)	−0.0060 (10)	0.0009 (10)	−0.0003 (11)
C16	0.0325 (11)	0.0325 (13)	0.0332 (12)	−0.0026 (10)	0.0073 (10)	0.0018 (10)
C17	0.0354 (12)	0.0422 (15)	0.0381 (13)	−0.0083 (11)	0.0046 (10)	0.0050 (12)
C18	0.0370 (13)	0.0574 (19)	0.0522 (16)	−0.0136 (13)	0.0069 (12)	0.0030 (14)
C19	0.0319 (12)	0.0470 (16)	0.0425 (14)	−0.0066 (11)	0.0004 (10)	0.0069 (12)
O1W	0.0450 (11)	0.0803 (16)	0.0830 (15)	−0.0032 (11)	−0.0032 (11)	0.0025 (12)

### Geometric parameters (Å, °)

**Table:**

Cu1—O1	1.8894 (16)	C6—C7	1.425 (3)
Cu1—O3	1.9494 (16)	C7—H7	0.9300
Cu1—N1	1.9582 (18)	C8—C9	1.518 (3)
Cu1—N2	1.9789 (18)	C8—C15	1.537 (3)
Cl1—C12	1.742 (3)	C8—H8	0.9800
N1—C7	1.297 (3)	C9—C10	1.392 (3)
N1—C8	1.489 (3)	C9—C14	1.401 (3)
N2—C17	1.318 (3)	C10—C11	1.383 (3)
N2—C19	1.378 (3)	C10—H10	0.9300
N3—C17	1.331 (3)	C11—C12	1.379 (3)
N3—C18	1.358 (3)	C11—H11	0.9300
N3—H3A	0.8600	C12—C13	1.379 (3)
O1—C1	1.296 (3)	C13—C14	1.381 (3)
O2—C16	1.236 (3)	C13—H13	0.9300
O3—C16	1.278 (3)	C14—H14	0.9300
C1—C6	1.419 (3)	C15—C16	1.507 (3)
C1—C2	1.420 (3)	C15—H15A	0.9700
C2—C3	1.362 (4)	C15—H15B	0.9700
C2—H2	0.9300	C17—H17	0.9300
C3—C4	1.393 (4)	C18—C19	1.344 (3)
C3—H3	0.9300	C18—H18	0.9300
C4—C5	1.360 (3)	C19—H19	0.9300
C4—H4	0.9300	O1W—H1W	0.8709
C5—C6	1.412 (3)	O1W—H2W1	0.8180
C5—H5	0.9300	O1W—H2W2	0.8325
			
O1—Cu1—O3	172.18 (7)	C9—C8—H8	106.6
O1—Cu1—N1	93.45 (7)	C15—C8—H8	106.6
O3—Cu1—N1	92.47 (7)	C10—C9—C14	117.2 (2)
O1—Cu1—N2	87.61 (7)	C10—C9—C8	120.8 (2)
O3—Cu1—N2	86.66 (7)	C14—C9—C8	121.8 (2)
N1—Cu1—N2	177.75 (8)	C11—C10—C9	121.9 (2)
C7—N1—C8	115.30 (18)	C11—C10—H10	119.1
C7—N1—Cu1	123.22 (15)	C9—C10—H10	119.1
C8—N1—Cu1	121.16 (14)	C12—C11—C10	119.2 (2)
C17—N2—C19	105.0 (2)	C12—C11—H11	120.4
C17—N2—Cu1	127.44 (16)	C10—C11—H11	120.4
C19—N2—Cu1	127.53 (16)	C11—C12—C13	120.8 (2)
C17—N3—C18	107.1 (2)	C11—C12—Cl1	119.5 (2)
C17—N3—H3A	126.5	C13—C12—Cl1	119.6 (2)
C18—N3—H3A	126.5	C12—C13—C14	119.4 (2)
C1—O1—Cu1	129.47 (15)	C12—C13—H13	120.3
C16—O3—Cu1	128.07 (16)	C14—C13—H13	120.3
O1—C1—C6	123.5 (2)	C13—C14—C9	121.5 (2)
O1—C1—C2	119.3 (2)	C13—C14—H14	119.2
C6—C1—C2	117.2 (2)	C9—C14—H14	119.2
C3—C2—C1	121.2 (2)	C16—C15—C8	114.26 (19)
C3—C2—H2	119.4	C16—C15—H15A	108.7
C1—C2—H2	119.4	C8—C15—H15A	108.7
C2—C3—C4	121.5 (2)	C16—C15—H15B	108.7
C2—C3—H3	119.2	C8—C15—H15B	108.7
C4—C3—H3	119.2	H15A—C15—H15B	107.6
C5—C4—C3	118.9 (2)	O2—C16—O3	122.0 (2)
C5—C4—H4	120.5	O2—C16—C15	119.9 (2)
C3—C4—H4	120.5	O3—C16—C15	118.1 (2)
C4—C5—C6	121.7 (2)	N2—C17—N3	111.7 (2)
C4—C5—H5	119.2	N2—C17—H17	124.1
C6—C5—H5	119.2	N3—C17—H17	124.1
C5—C6—C1	119.5 (2)	C19—C18—N3	106.9 (2)
C5—C6—C7	118.2 (2)	C19—C18—H18	126.6
C1—C6—C7	122.2 (2)	N3—C18—H18	126.6
N1—C7—C6	128.1 (2)	C18—C19—N2	109.3 (2)
N1—C7—H7	115.9	C18—C19—H19	125.4
C6—C7—H7	115.9	N2—C19—H19	125.4
N1—C8—C9	112.78 (18)	H1W—O1W—H2W1	119.3
N1—C8—C15	109.03 (17)	H1W—O1W—H2W2	115.1
C9—C8—C15	114.66 (19)	H2W1—O1W—H2W2	106.8
N1—C8—H8	106.6		

### Hydrogen-bond geometry (Å, °)

**Table:**

*D*—H···*A*	*D*—H	H···*A*	*D*···*A*	*D*—H···*A*
O1W—H1W···O2^i^	0.87	1.98	2.799 (3)	156
O1W—H2W1···O1W^ii^	0.82	2.01	2.826 (4)	172
O1W—H2W2···O1W^iii^	0.83	2.02	2.822 (5)	163
N3—H3A···O2^iv^	0.86	1.90	2.758 (3)	172

Symmetry codes: (i) −*x*+1/2, −*y*+3/2, −*z*; (ii) −*x*+1, −*y*+2, −*z*; (iii) −*x*+1, −*y*+1, −*z*; (iv) −*x*+1/2, −*y*+5/2, −*z*.
